# Differentiating origins of outflow tract ventricular arrhythmias: a comparison of three different electrocardiographic algorithms

**DOI:** 10.1590/1414-431X20165206

**Published:** 2016-04-29

**Authors:** Z.Y. Jiao, Y.B. Li, J. Mao, X.Y. Liu, X.C. Yang, C. Tan, J.M. Chu, X.P. Liu

**Affiliations:** 1 The Heart Center, Beijing Chao-Yang Hospital, Capital Medical University, Beijing, China; 2 Department of Cardiology, FuWai Hospital, CAMS and PUMC, Beijing, China; 3 Department of Cardiology, The Military General Hospital of Beijing PLA, Beijing, China

**Keywords:** Premature ventricular contraction, Ventricular tachycardia, Electrocardiogram, Radiofrequency catheter ablation

## Abstract

Our objective is to evaluate the accuracy of three algorithms in differentiating the origins of outflow tract ventricular arrhythmias (OTVAs). This study involved 110 consecutive patients with OTVAs for whom a standard 12-lead surface electrocardiogram (ECG) showed typical left bundle branch block morphology with an inferior axis. All the ECG tracings were retrospectively analyzed using the following three recently published ECG algorithms: *1*) the transitional zone (TZ) index, *2*) the V_2_ transition ratio, and *3*) V_2_ R wave duration and R/S wave amplitude indices. Considering all patients, the V_2_ transition ratio had the highest sensitivity (92.3%), while the R wave duration and R/S wave amplitude indices in V_2_ had the highest specificity (93.9%). The latter finding had a maximal area under the ROC curve of 0.925. In patients with left ventricular (LV) rotation, the V_2_ transition ratio had the highest sensitivity (94.1%), while the R wave duration and R/S wave amplitude indices in V_2_ had the highest specificity (87.5%). The former finding had a maximal area under the ROC curve of 0.892. All three published ECG algorithms are effective in differentiating the origin of OTVAs, while the V_2_ transition ratio, and the V_2_ R wave duration and R/S wave amplitude indices are the most sensitive and specific algorithms, respectively. Amongst all of the patients, the V_2_ R wave duration and R/S wave amplitude algorithm had the maximal area under the ROC curve, but in patients with LV rotation the V_2_ transition ratio algorithm had the maximum area under the ROC curve.

## Introduction

The outflow tract ventricular arrhythmia (OTVA) is a common medical condition, and approximately 80% of cases originate from the right ventricular outflow tract (RVOT) (1). There are three clinical forms of OTVA manifestation: 1) paroxysmal sustained monomorphic ventricular tachycardia, 2) repetitive nonsustained ventricular tachycardia or 3) premature ventricular contractions (PVCs). Radiofrequency (RF) catheter ablation, which has a high success rate, is currently the preferred therapy for OTVA (2,3) in symptomatic patients and/or in patients with failure of anti-arrhythmic drug therapy (e.g., beta-blocker, sodium channel blockers), and in patients with a decline in left ventricular (LV) function due to OTVA burden. Surgical approaches for OTVAs differ depending on the origin of the arrhythmia. Therefore, differentiating the origins of OTVAs based on the findings of a surface electrocardiogram (ECG) shortens the operative time and reduces unnecessary punctures. Based on the literature, three different electrocardiographic algorithms differentiate the origins of left and right OTVAs. However, the accuracies of the three algorithms have never been compared. Thus, we have retrospectively analyzed cases involving successful ablation, and compared the reliabilities of the three algorithms in distinguishing OTVA origins.

## Material and Methods

### Data collection

One hundred and ten (51 males and 59 females) consecutive patients (mean age, 45.3±15.3) with PVCs or ventricular tachycardia (VT) who underwent successful ablation at one of the three Third-Class-A Hospitals of the study were enrolled. All the ablation targets were located in the outflow tract, and patients who underwent successful ablation via large veins were excluded. One patient failed two RF treatments due to PVCs. Standard 12-lead ECGs were obtained from all patients under sinus rhythm (SR) and OTVA conditions. All the OTVA ECGs showed a typical left bundle branch block morphology with an inferior axis. Holter monitoring revealed 10,000 monomorphic PVCs/24 h or PVC loads ≥10%. Structural heart diseases were excluded by echocardiography, cardiovascular CT, or coronary angiography (for some patients). Patients discontinued anti-arrhythmic drugs (AADs) for at least five half-lives pre-operatively. All patients signed informed consents pre-operatively. Of the 110 patients, 84 (76.4%) underwent RF ablation targeted at the RVOT via the femoral venous approach, and 26 (23.6%) had RF ablations targeted at the aortic sinus cusp (ASC) via the femoral arterial approach.

All procedures and protocols were approved by the Institutional Beijing Chao-Yang Hospital Ethics Committee, Capital Medical University, Beijing, China.

### ECG analysis

During OTVA conditions, all standard 12-lead surface ECGs showed complete left bundle branch block with an inferior axis. Software (DatInf^¯^ Measure, Germany) was used to measure the values of QRS waves under SR and OTVA conditions on the same ECG; for monomorphic PVCs, only the first QRS was measured. The following 3 indices were sequentially calculated.

*Transitional zone (TZ) index* (4). The chest leads involved in the TZ are the ones with a R/S wave ratio of 0.9-1.1. The number of the lead is the score. If the TZ is located between two leads, the score is then calculated by adding 0.5 to the number of the previous lead. For example, if the TZ is on the V3 lead, the score is 3; if the TZ appeared between the V3 and V4 leads, the score is 3.5. The TZ index is defined using the TZ score under OTVA conditions minus the TZ score under SR conditions. An ASC origin is indicated by a TZ index <0, while an RVOT is suggested by a TZ index ≥0.

*V*_2_ *transition ratio* (5). An ASC origin is indicated by a (R/R+S)_OTVA_-to-(R/R+S)_SR_ ratio ≥0.5, while an RVOT origin is suggested by a (R/R+S)_OTVA_-to-(R/R+S)_SR_ ratio <0.5.

**Figure 1:**
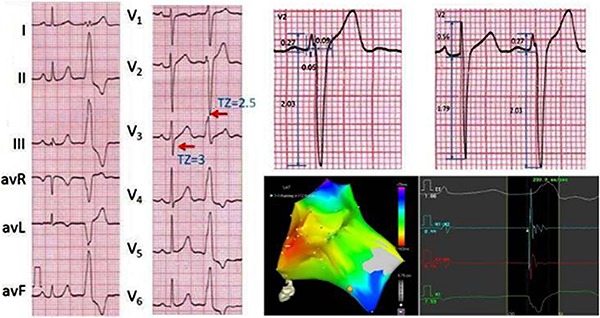
Electrocardiogram results used for differentiation of the origins of outflow tract ventricular arrhythmias (OTVAs) using three algorithms: transitional zone (TZ) index; V_2_ transition ratio; and V_2_ R wave duration and R/S wave amplitude indices. For this representative patient, the premature ventricular contractions transitional zone (TZ) score is 2.5, the sinus rhythm TZ score is 3, and the TZ index is -0.5, indicating an aortic sinus cusp (ASC) origin OTVA. V_2_ transition ratio was calculated as (R/R+S)_OTVA_/(R/R+S)_SR_ = (0.27/2.03+0.27)/(0.56/1.79+0.56) = 0.73. V_2_ R wave duration index = V_2_ R wave duration/ duration from the starting point of QRS in V_4_ to the ending point of QRS in avF = 0.05/0.14 = 0.35; R/S wave amplitude index = 0.27/2.03 = 0.13, confirming the ASC origin OTVA. The intraoperative target is located at the posterior septum of the right ventricular outflow tract.

*V*_2_ *R wave duration and R/S wave amplitude indices* (6). This refers to the R wave duration in the V_1_ or V_2_ lead in relation to the entire QRS wave duration (R/QRS). An ASC origin is indicated if the R/QRS wave duration ratio is ≥50%. The R/S wave amplitude refers to the R/S wave amplitude ratio. An ASC origin is indicated if the R/S ratio is ≥30% (Figure 1). Normally, R/S ratios are similar in the V_3_ and V_4_ leads. A counterclockwise rotation is indicated if RS waves appear in the V_1_ and V_2_ leads. Similarly, a clockwise rotation is suggested if RS waves appear in the V_5_ and V_6_ leads.

### Mapping and ablation

The right femoral vein was routinely cannulated. A 3.5-mm open-tip irrigated catheter (NaviStar Thermocool™; Biosense-Webster Inc., USA) was placed at the right ventricular outflow tract, adopting activation mapping and pace mapping to determine the ablation target. If an ideal target was not pursued in the right ventricle another catheter was inserted via the right femoral artery mapping at the left ventricular outflow tract. The ablation settings were as follows: power of 20-25 W, upper temperature limit of 43°C, perfusion flow rate of 30-50 mL/h, and discharge duration of 30-60 s. No thromboses or hemorrhage occurred during a single procedure.

### Criteria for success

The criterion for immediate success after RF ablation was the absence of PVCs or the absence of PVC symptoms observed 30 min after intravenous infusion of isoproterenol.

The criterion for long-term success was absence of clinical episodes of ventricular arrhythmias during a 1-year follow-up (outpatient or by telephone call).

### Statistical analysis

SPSS18.0 (SPSS Inc., USA) and STATA11.0 (StataCorp., USA) were used for statistical analyses. Data are reported as means±SD. The sensitivity, specificity, positive predictive value, and negative predictive value of each indicator were calculated using the Fisher's exact test with a 4-fold table. The receiver operating characteristic (ROC) curve analysis was used to assess the three algorithms. P<0.05 indicated statistical significance.

## Results

Of the 84 RVOT cases, 72 (86%) were RF ablations that targeted the septum and 12 (14%) targeted the free way. Of the 26 ASC cases, 1 (4%) was a RF ablation that targeted the right coronary sinus, 22 (88%) targeted the left coronary sinus, and 2 (8%) targeted the region between the two sinuses.

No significant differences were detected with respect to the clinical information of the patients with RVOT and ASC origins, including gender, age, PVC load, left ventricular end-systolic and end-diastolic diameters, wall thickness, and left ventricular ejection fraction (Table 1). There were 66 patients in the RVOT group and 20 patients in the ASC group taking AADs pre-operatively; the difference between the two groups was not significant. Under SR conditions, rotations appeared in precordial ECGs of 31 (36%) patients from the RVOT group; of the 31 patients, 19 were clockwise rotations and 21 were counterclockwise rotations. Eleven patients from the ASC group had cardiac rotations, of which 5 were counterclockwise rotations and 6 were clockwise rotations. No significant difference was found in cardiac rotations between the two groups.

**Table:**
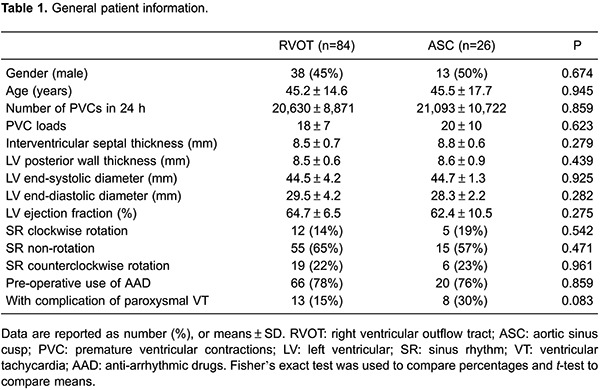

The ECG characteristics of the patients are shown in Table 2. Significant differences were found in the TZ index, V_2_ transition ratio, and V_2_ R wave duration and R/S wave amplitude indices of the two groups; no significant differences were found in the SR TZ score, V_2_ R wave amplitude, and PVC QRS duration.

**Table:**
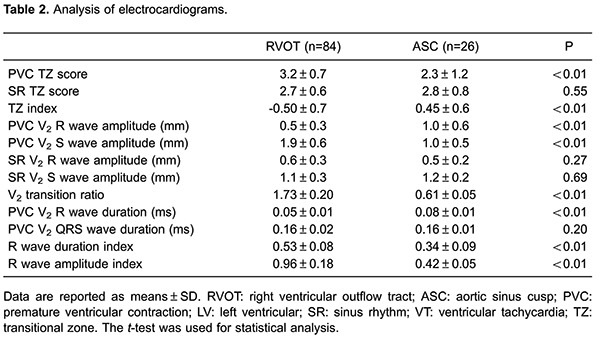

**Figure 2:**
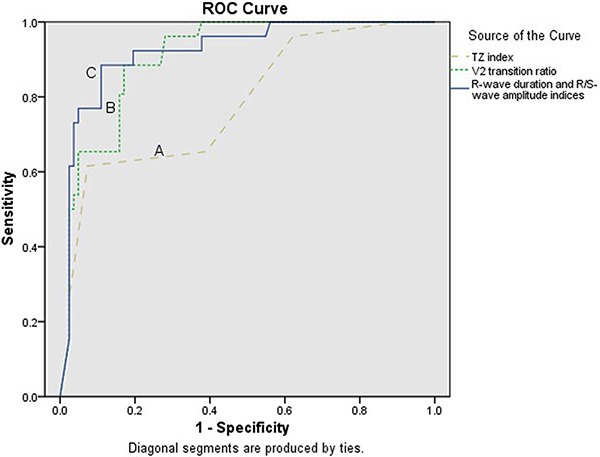
AUCs of the three algorithms in all patients. AUC *A*: transitional zone (TZ) index=0.84, *B*: V_2_ transitional ratio=0.91, *C*: V_2_ R wave duration and R/S wave amplitude indices = 0.925. Pairwise comparison: *A* and *B* (P=0.0082), *A* and *C* (P=0.0051), *B* and *C* (P=0.4711).

The V_2_ transition ratio had the highest sensitivity (92.3%; Table 3), while the R wave duration and R/S wave amplitude indices in V_2_ had the highest specificity (93.9%). The latter finding had a maximal AUC of 0.925 (Figure 2), followed by the V_2_ transition ratio (0.91) and the TZ index (0.84) (P<0.01).

**Table:**
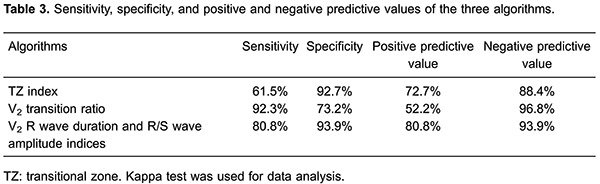

**Figure 3:**
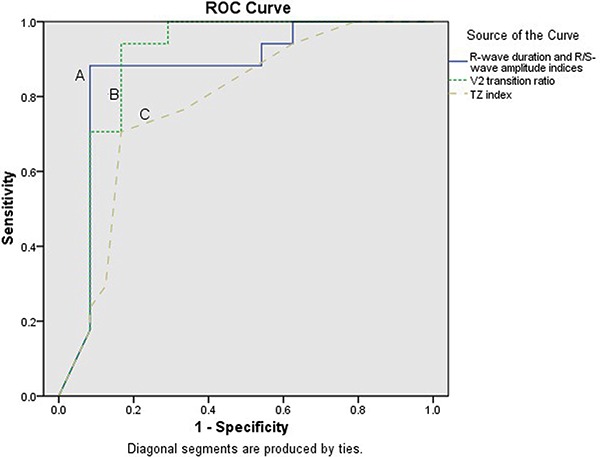
AUCs of the three algorithms in patients with rotation. AUC *A*: V_2_ R wave duration and R/S wave amplitude indices = 0.86; *B*: V_2_ transitional ratio = 0.89; *C*: TZ index = 0.78 (P=0.1416).

In patients with LV rotation, the V_2_ transition ratio had the highest sensitivity (94.1%; Table 4), while the R wave duration and R/S wave amplitude indices in V_2_ had the highest specificity (87.5%). The former finding had a maximal AUC of 0.892 (Figure 3), followed by the V_2_ R wave duration and R/S wave amplitude indices (0.865) and TZ index (0.781) (P>0.05).

**Table:**
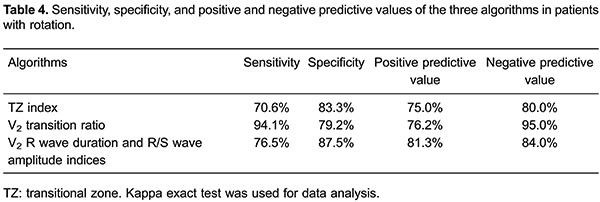

## Discussion

Several reports have focused on differentiating the origins of PVCs with pre-operative ECGs and some reports have proposed systematic procedures for demonstrating this process (7-11). Recently, reports involving patients with ECG findings indicating rotation under SR conditions have presented novel differentiating algorithms based on the V_2_ transition ratio and TZ index. A comparison between these two algorithms and the previous approaches has not been reported, which served as the basis for conducting the current study. Of the three algorithms, the V_2_ transition ratio had the highest sensitivity, while the R wave duration and R/S wave amplitude indices in V_2_ had the highest specificity. Analyses of the AUC of the 3 algorithms, which takes sensitivity and specificity into account, were all between 0.7 and 0.9; therefore, all 3 algorithms seem to be reliable approaches to differentiating the origins of OTVAs. In spite of the presence or absence of cardiac rotation, no significant differences existed in AUCs of the V_2_ transition ratio and V_2_ R wave duration and R/S wave amplitude indices, which indicated that these two algorithms had similar diagnostic values in OTVAs. The TZ index had a diagnostic value similar to the V_2_ transition ratio in patients with rotation.

Of the 3 algorithms, the TZ index was slightly inferior. Because the score was calculated at an advancement of 0.5, subtle differences among the leads might be neglected. It is possible that there were 2 leads whose R/S wave ratios were between 0.9 and 1.1 simultaneously, making it difficult to determine the score and therefore affecting the accuracy of target positioning. As a commonly observed phenomenon in a clinical ECG, cardiac rotation is affected by many factors (12). Sengupta et al. (13) defined the term cardiac rotation, as a circumferential motion of the LV around the longitudinal axis. At the early stage of isovolumetric contraction, mild clockwise rotation takes place in the LV apex, and at the ejection period it turns into a counterclockwise rotation. Motions of the LV base are opposite to those of the LV apex. Compared with the rotation degree of the apex, that of the base is significantly lower (13,14). In non-invasive inspections performed nowadays, myocardial tissue tagging by myocardial resonance is the gold standard for LV rotation assessment (15). Rotation assessment of the patients in this study have only been obtained by ECGs, therefore further confirmation by myocardial resonance for myocardial tagging would be required. However, rotation amplitude of the base was lower than that of the apex. Therefore, the mild rotations of the base in these patients did not affect the determination of the OTVA targets.

In summary, our study showed that, in spite of the presence or absence of cardiac rotation, the V_2_ transition ratio had the highest sensitivity, while the R wave duration and R/S wave amplitude indices in V_2_ had the highest specificity. No significant differences were found in AUCs of the two algorithms, which indicates similar diagnostic values in differentiating the origins of OTVAs, while the TZ index was slightly inferior. No significant differences in diagnostic values were found among the three algorithms for patients with rotations in the precordial leads. These algorithms are currently used in clinical practice, each with its own advantages. Applying two or three methods to define the OTVAs origin with mutual verification may thus be recommended.

A shortcoming of this research was that the data were collected from three hospitals. Therefore, the lead positions of the ECGs might have been different. The V_2_ lead was chosen because this position is relatively reliable. This retrospective study has verified the accuracy of different algorithms used in identifying the origins of OTVAs, through follow-up evaluations. Although being one of the studies with the largest sample size to date, a prospective study should be conducted for verification.
